# Osimertinib‐induced BRAF mutation in a single metastatic lesion among multiple pulmonary lesions in a case of lung cancer with EGFR exon 19 deletion

**DOI:** 10.1002/rcr2.70003

**Published:** 2024-08-13

**Authors:** Hiroyuki Miura, Jun Miura, Shinichi Goto, Tomoko Yamamoto

**Affiliations:** ^1^ Department of Thoracic Surgery Akiru Municipal Medical Centre Tokyo Japan; ^2^ Department of Surgery Kyorin University School of Medicine Tokyo Japan; ^3^ Department of Respirology Akiru Municipal Medical Centre Tokyo Japan; ^4^ Department of Pathology Tokyo Women's Medical University Tokyo Japan

**Keywords:** BRAF V600E, EGFR‐TKI, lung cancer, osimertinib, resistance mechanism

## Abstract

One of the resistant mechanisms of EGFR‐TKIs is BRAF V600E mutation. Herein, we present the case of a 54‐year‐old Japanese female who underwent a right middle lobectomy for pathological stage IIB lung adenocarcinoma. One year and nine months after the surgery, she developed multiple intrapulmonary metastases. Osimertinib was administered due to EGFR exon 19 deletion. Although all intrapulmonary metastases had shrunk, the nodule at the superior segment of left lung enlarged after postoperative 4 years. The tumour was resected and BRAF V600E mutation and exon 19 deletion were detected. Three months after treatment with dabrafenib and trametinib instead of osimertinib, the remaining intrapulmonary metastases increased again. The continued growth of the metastatic foci even after EGFR‐TKI may indicate an acquired resistance. Thus, a repeat biopsy will aid in confirming the new gene expression. It should have been necessary to administer an additional dose of dabrafenib and trametinib without discontinuing osimertinib.

## Graphical abstract

Osimertinib is effective for treating patients with lung cancers with sensitive epidermal growth factor receptor (EGFR) mutations. However, resistance mechanisms have been reported. Herein, we report a case of BRAF V600E mutation in only one of the multiple intrapulmonary metastatic lesions during treatment with osimertinib for patients with lung adenocarcinoma with EGFR exon 19 deletion.

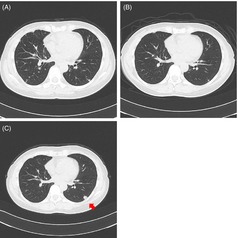

## INTRODUCTION

Osimertinib is effective for treating patients with lung cancers with sensitive epidermal growth factor receptor (EGFR) mutations. However, resistance mechanisms such as MET amplification, secondary mutations such as C797X mutation, PIK3CA amplification, HER2 amplification, and transformation to small cell lung cancer have been reported.^1^ BRAF V600E mutation is also an acquired resistance mechanism in lung cancer.^1^ If acquired resistance does occur, it is questionable whether all metastatic lesions will acquire resistance. Herein, we report a case of BRAF V600E mutation in only one of the multiple intrapulmonary metastatic lesions during treatment with osimertinib for patients with lung adenocarcinoma with EGFR exon 19 deletion.

## CASE REPORT

A 54‐year‐old non‐smoking woman presented with an abnormal shadow on a chest x‐ray during an annual check‐up. Her medical history was notable only for hypertension. Her family history was not remarkable. Under the diagnosis of lung cancer by trans bronchial brushing cytology, a right middle lobectomy and mediastinal lymph node dissection were performed. The tumour, measuring 19 × 19 × 22 mm, proved to be a pT1bN1M0 papillary adenocarcinoma with hilar lymph node involvement. The single gene analysis showed EGFR exon 19 deletion in the resected tumour but negative for BRAF V600E. The patient underwent four courses of postoperative adjuvant chemotherapy with cisplatin and vinorelbine.

**FIGURE 1:**
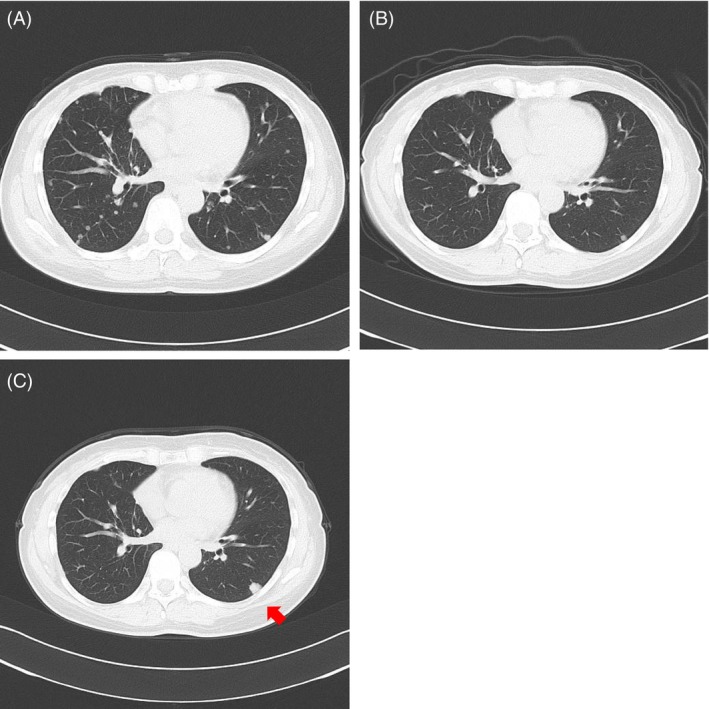
Chest computed tomography obtained (A) 1 year and 9 months, (B) 3 years and 2 months, and (C) 4 years (arrow, enlarged S6 nodule) after lobectomy.

One year and nine months after the surgery, multiple intrapulmonary metastases were identified in both lungs (Figure 1A). Thus, molecularly targeted therapy using osimertinib was initiated considering the presence of EGFR exon 19 deletion. Three years and two months after the surgery, all intrapulmonary metastases had disappeared or shrunk (Figure 1B). However, 4 years after the surgery, the nodule at the superior segment (S6) of left lung enlarged from 6 to 11 mm (Figure 1C). The nodule was resected using VATS. Histopathological examination of the excised lesion revealed papillary adenocarcinoma that resembled the primary tumour. The single gene analysis was performed again to the metastatic tumour.

Upon comparing the single gene analysis results and tumour progressive score between the primary and the resected metastatic tumour, we observed EGFR exon 19 deletion in the both tumour but BRAF V600E mutations only in metastatic tumour (Table 1). The entire tumour was considered to have acquired resistance, so dabrafenib and trametinib were started while osimertinib was stopped. Three months after the treatment with dabrafenib and trametinib, the remaining intrapulmonary metastases had increased again. Thus, osimertinib was resumed instead of dabrafenib and trametinib, resulting in the diminution of all the metastatic lung tumours. Nonetheless, brain metastases were observed 5 years and 2 months after the initial surgery. A combination of chemotherapy and immune checkpoint inhibitor, consisting of carboplatin, pemetrexed and pembrolizumab, were administered to the patient (Table 2).

**TABLE 1:** Comparison between the primary and metastatic tumour characteristics.

Characteristics	Primary tumour	Metastatic lesion
EGFR (ex19)	+	+
ALK	−	−
ROS‐1	−	−
BRAF V600E	−	+
MET	−	−
TPS (%)	0	5

Abbreviation: TPS, tumour progression score.

**TABLE 2:** Clinical course of the patient.

Day	Event	Treatment
X	Right middle lobectomy + ND2a‐2	CDDP + VNR— 4 courses
1y 9 m	Multiple pulmonary metastases	Osimertinib
4y	S6 nodule enlargement for which left S6 partial resection was performed	Dabrafenib + trametinib
4y 6 m	Pulmonary metastatic lesion enlargement	Osimertinib
5y	Brain metastasis	γ‐knife CBDCA + Pemetrexed + Pembrolizumab

Abbreviations: CBDCA, carboplatin; CDDP, cisplatin; VNR, vinorelbine.

## DISCUSSION

BRAF V600E mutations are found in approximately 3% of adenocarcinomas and are mutually exclusive with other driver genes.^2^ According to the multi‐institutional lung cancer genomic screening project (LC‐SCRUM‐Asia), BRAF mutations are detected in 3.5% of all non‐small cell lung cancer cases. Among these, 31% are BRAF V600E mutations, for which dabrafenib and trametinib are effective. BRAF V600E mutations have also been reported as one of the resistance mechanisms of EGFR‐tyrosine kinase inhibitor (TKI) therapy. However, it is unclear whether all metastatic lesions acquire the BRAF V600E mutation.

In our patient, all intrapulmonary metastases, including the left S6 tumour, had decreased in size with osimertinib treatment. However, the intrapulmonary metastases increased in size during dabrafenib and trametinib treatment after S6 tumour removal. Therefore, all metastatic tumours, except the left S6 tumour, were considered to be without a BRAF V600E mutation.

There are several reports of lesions exhibiting both EGFR mutations and BRAF V600E mutations.^3^, ^4^, ^5^ However, there are few reports of a single lesion among multiple lung metastatic lesions with EGFR exon 19 deletion demonstrating BRAF V600E mutation.

Cells with a BRAF mutation could exist rarely in a chimeric state in the primary tumour. The recent multi‐institutional genomic sturdy demonstrated that EGFR can exist concurrently with a BRAF mutation (*n* = 17/380; 4%).^3^ In our case, BRAF V600E was not expressed in the primary tumour, and rather than assuming that the slight simultaneous cells with BRAF V600E and EGFR ex19 deletion, metastasized to only one location, it is more consistent to assume that only one of the multiple lung tumours acquired resistance, given that the S6 tumour shrank once and then regressed.

Osimertinib is effective against other metastatic lesions. Therefore, it was important to continue osimertinib even when dabrafenib and trametinib are added, in the setting of acquired resistance to osimertinib as the original driver EGFR mutation is coexisting with BRAF V600E. Meng et al. reported a case of lung adenocarcinoma with EGFR exon 19 deletion and a T790M mutation, which was treated with osimertinib. Although the patient acquired a BRAF V600 mutation, clinical response was observed for 13.4 months with the concurrent use of dabrafenib and trametinib with osimertinib.^4^ Wei et al. reported the treatment outcome of patients with advanced EGFR‐mutant non‐small cell carcinoma with concomitant BRAF variations. The median progression‐free survival of patients who were treated with chemotherapy was lower than that of those treated with EGFR and BRAF co‐inhibitory drugs (5.0 vs. 7.8 months).^5^

In conclusion, growth of a metastatic foci in the setting of an otherwise effective EGFR‐TKI regimen may indicate an acquired resistance mechanism. In such patients, aggressive repeat biopsy is useful to confirm the new gene expression.

## AUTHOR CONTRIBUTIONS

Dr. Hiroyuki Miura and Dr. Shinichi Goto helped in the conception and design of the work and the acquisition and analysis or interpretation of data for the work. Dr. Jun Miura drafted the work and revised it critically for important intellectual content. Dr. Yamamoto diagnosed this tumour pathologically. All authors contributed to the final version of this manuscript and approved it for publication.

## CONFLICT OF INTEREST STATEMENT

The authors declare no conflicts of interest.

## ETHICS STATEMENT

The authors declare that appropriate written informed consent was obtained for the publication of this manuscript and accompanying images.

## Data Availability

The data that support the findings of this study are available from the corresponding author upon reasonable request.
